# Multivalent display of the antimicrobial peptides BP100 and BP143

**DOI:** 10.3762/bjoc.8.237

**Published:** 2012-12-03

**Authors:** Imma Güell, Rafael Ferre, Kasper Kildegaard Sørensen, Esther Badosa, Iteng Ng-Choi, Emilio Montesinos, Eduard Bardají, Lidia Feliu, Knud J Jensen, Marta Planas

**Affiliations:** 1 LIPPSO, Department of Chemistry, University of Girona, Campus Montilivi, 17071 Girona, Spain; 2 IGM, Faculty of Life Sciences University of Copenhagen, DK-1871 Frederiksberg, Denmark; 3 Laboratory of Plant Pathology, Institute of Food and Agricultural Technology-CIDSAV-CeRTA, University of Girona, Campus Montilivi, 17071 Girona, Spain

**Keywords:** antimicrobial activity, carbopeptides, multimeric structures, oxime ligation, phytopathogenic bacteria

## Abstract

Carbohydrates are considered as promising templates for the display of multiple copies of antimicrobial peptides. Herein, we describe the design and synthesis of chimeric structures containing two or four copies of the antimicrobial peptides KKLFKKILKYL-NH_2_ (**BP100**) and KKLfKKILKYL-NH_2_ (**BP143**) attached to the carbohydrate template cyclodithioerythritol (cDTE) or α-D-galactopyranoside (Gal*p*). The synthesis involved the preparation of the corresponding peptide aldehyde followed by coupling to an aminooxy-functionalized carbohydrate template. After purification, the multivalent display systems were obtained in high purities (90–98%) and in good yields (42–64%). These compounds were tested against plant and human pathogenic bacteria and screened for their cytotoxicity on eukaryotic cells. They showed lower MIC values than the parent peptides against the bacteria analyzed. In particular, the carbopeptides derived from cDTE and Gal*p*, which contained two or four copies of **BP100**, respectively, were 2- to 8-fold more active than the monomeric peptide against the phytopathogenic bacteria. These results suggest that preassembling antimicrobial peptides to multimeric structures is not always associated with a significant improvement of the activity. In contrast, the carbopeptides synthesized were active against human red blood cells pointing out that peptide preassembly is critical for the hemolytic activity. Notably, peptide preassembly resulted in an enhanced bactericidal effect.

## Introduction

The emergence of antibiotic resistance is a major problem in human health as well as in agronomy and there is a considerable current interest in developing novel antimicrobial compounds [1–2]. In recent years, a growing number of studies have shown that antimicrobial peptides constitute a potential alternative to traditional antibiotics. To date, a very large number of antimicrobial peptides have been produced based on naturally occurring products or by de novo design, and most of them display a broad spectrum of antimicrobial activities [3–4]. Despite their remarkable structural diversity, these peptides share several characteristics: the sequence length is typically between 12 to 59 amino acids, they bear a positive charge of +2 to +9, and they show an amphipathic character. The electrostatic interaction of antimicrobial peptides with membranes is a key factor in determining their biological activity [1,3,5–8]. For many of these peptides, membrane disruption is considered the primary mechanism of cell death. Although a general understanding has been achieved, the precise mechanism of peptide–membrane interaction and cell killing has not been firmly established for many peptides. Several models have been proposed to account for the morphologic changes in the membrane induced by antimicrobial peptides, such as pore formation, lysis or peptide translocation into the cytoplasm. Either model relies on relatively high local density and synergy of monomeric peptides.

It has occasionally been reported that an improved binding to microbial membranes and thus an improved antimicrobial activity can be achieved if several copies of an antimicrobial peptide are linked together to form multimeric species [9–15]. The hypothesis of the assembly model is based on prenucleating a number of appropriately spaced and oriented antimicrobial peptide monomers in order to reduce the threshold concentration required for their activity. The interaction of these multivalent species should a priori make the interaction with the microbial membrane strongly and entropically more favorable. Representative studies include multimeric derivatives of alamethicin [16] and the dimeric antimicrobial peptide A3-APO [17]. Moreover, studies of multivalent antimicrobial peptides are of growing importance because they could represent an interesting tool to provide insights into the modes of action of antimicrobial peptides, which are generally poorly understood.

Several scaffolds, such as linear and cyclic peptides, alkyne-functionalized dendrimers, a branched lysine core, and also a polymaleic polymer, have been exploited as templates for the synthesis of multivalent antimicrobial peptides [18–22]. Carbohydrates are promising candidates as templates for the display of functional groups due to their inherent multifunctionality, the relative rigidity of their structure, and the ease of regioselective chemical manipulation [23–25]. Jensen and co-workers reported the synthesis of carbopeptides and carboproteins bearing several copies of a peptide or a protein tethered in a carbohydrate template. They developed an efficient strategy for the synthesis of these carboproteins, in which C-terminal peptide aldehydes are ligated by oxime bond formation to aminooxyacetyl-functionalized monosaccharide templates, such as cyclodithioerythritol (cDTE) or α-D-galactopyranoside (Gal*p*) [23,26–27].

**Figure 1:**
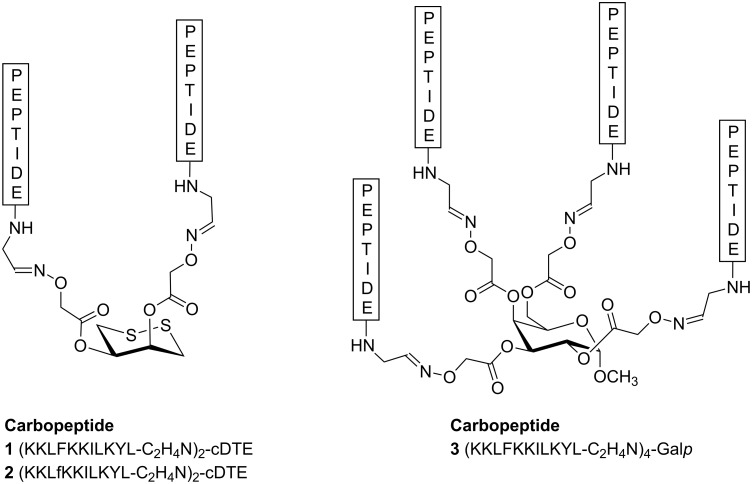
Structure of the carbopeptides **1**–**3**.

Here we describe the use of carbohydrate templates for the display of multiple copies of antimicrobial peptides. Our aim in this study was to evaluate whether the assembly of cecropin A-melittin antimicrobial undecapeptide hybrids on a carbohydrate template could provide carbopeptides with improved biological properties. In particular, KKLFKKILKYL-NH_2_ (**BP100**) and KKLfKKILKYL-NH_2_ (**BP143**) were selected based on their high antibacterial activity against the plant pathogenic bacteria *Erwinia amylovora*, *Xanthomonas axonopodis* pv. *vesicatoria* and *Pseudomonas syringae* pv. *syringae* [28–29]*. ***BP100** displayed minimum inhibitory concentration (MIC) values in the range 2.5–7.5 μM and also showed low hemolysis (22% at 150 μM). **BP143**, which contains one D-amino acid, was as active, significantly less hemolytic (2% at 150 μM), and more stable to protease degradation than **BP100**. Thus, carbopeptides **1**–**3** were designed and synthesized by linking two or four copies of **BP100** and **BP143** on a cDTE or a Gal*p* template (Figure 1). The antibacterial and hemolytic activities and the bactericidal effect of the carbopeptides **1**–**3** were analyzed and will be discussed.

## Results

### Design and synthesis of the carbopeptides

Two-stranded and four-stranded carbopeptides **1**–**3** were designed by coupling of two or four copies of a C-terminal undecapeptide aldehyde to an aminooxy-functionalized carbohydrate template (Figure 1). The previously reported cyclodithioerythritol (cDTE) was used as a template for the two-stranded carbopeptides **1** and **2** [23,26–27]. The α-D-galactopyranoside (Gal*p*) template was required to obtain the four-stranded carbopeptide **3**. The sequence of the peptide aldehydes was based on the peptides KKLFKKILKYL-NH_2_ (**BP100**) and KKLfKKILKYL-NH_2_ (**BP143**), which have been shown to display high antibacterial activity [28–29].

**Scheme 1:**
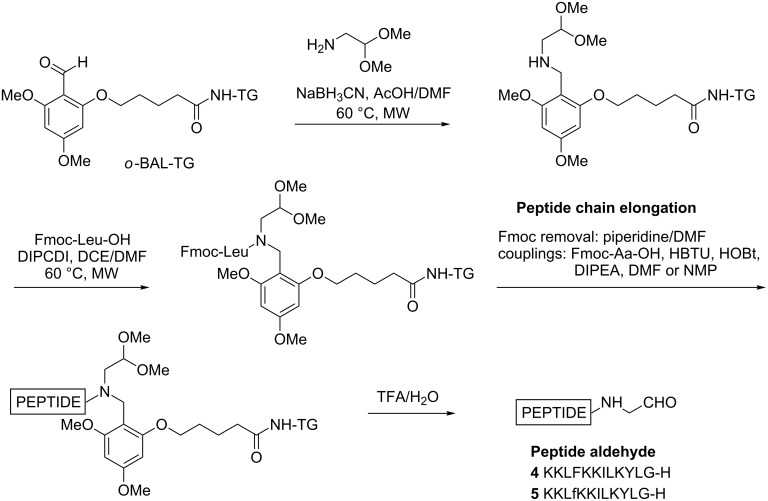
Solid-phase synthesis of the peptide aldehydes **4** and **5**.

Templates cDTE and Gal*p* functionalized with aminooxyacetic acid were prepared as before [23,30]. Peptide aldehydes KKLFKKILKYLG-H (**4**) and KKLfKKILKYLG-H (**5**), derived from **BP100** and **BP143**, respectively, were prepared by using a standard backbone amide linker (BAL) strategy (Scheme 1). PALdehyde was coupled onto an amino-functionalized TentaGel (TG) resin followed by reductive amination with aminoacetaldehyde dimethyl acetal, which served as a precursor for the C-terminal glycinal residue. Next, acylation of the secondary nitrogen with Fmoc-Leu-OH was mediated by *N*,*N*’-diisopropylcarbodiimide (DIPCDI) with microwave heating at 60 °C. Peptide chain elongation followed standard 9-fluorenylmethoxycarbonyl (Fmoc) solid-phase chemistry. After chain assembly was completed, peptides were released from the support by treatment with trifluoroacetic acid (TFA), which removed the *tert*-butyl (*t*-Bu)/*tert*-butyloxycarbonyl (Boc) groups and also hydrolyzed the acetal to the required aldehyde. When the cleavage was performed by using TFA/triisopropylsilane (TIS)/H_2_O (95:2.5:2.5), the corresponding peptide alcohol was obtained. It has been previously reported that silanes under acidic conditions can promote the reduction of aldehydes [27]. Cleavage of the peptidyl resins with TFA/H_2_O (95:5) followed by purification by reversed-phase high-performance liquid chromatography (RP-HPLC) afforded peptide aldehydes **4** and **5** in 99% purity, and their structure was confirmed by electrospray-ionization mass spectrometry (ESIMS).

**Scheme 2:**
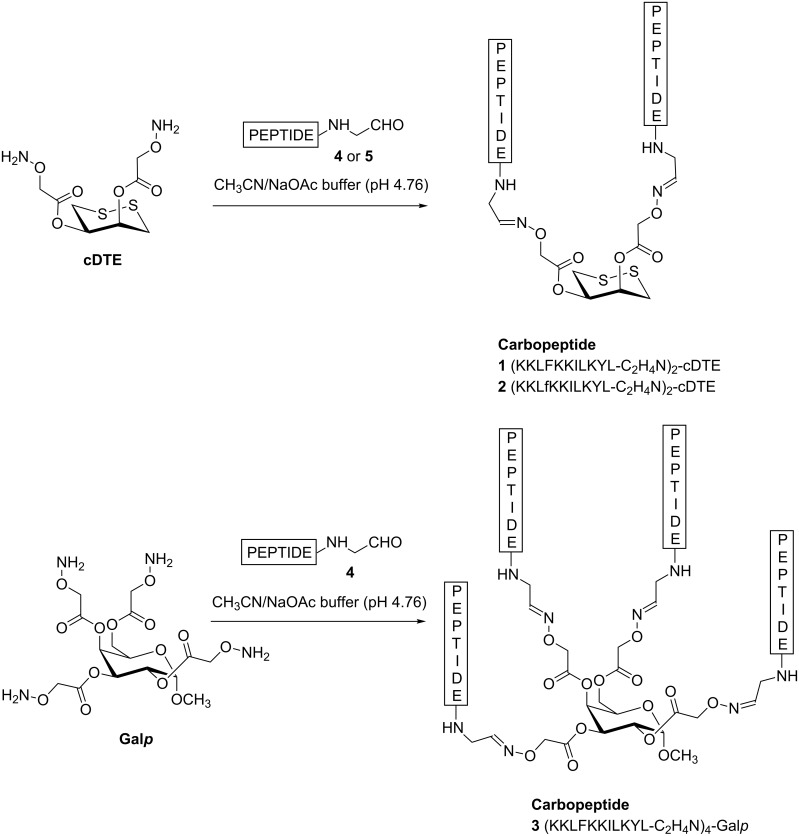
Synthesis of the carbopeptides **1**–**3**.

With templates and peptide aldehydes in hand, the four-stranded structure **3** was prepared from Gal*p* and peptide aldehyde **4** (Scheme 2). First, the reaction was conducted in a 1:1 solution of CH_3_CN and acetate buffer (0.1 M, pH 4.76) at room temperature for 2 h, but the expected carbopeptide **3** was not obtained. Since the use of aniline as nucleophilic catalyst for oxime ligation has been described to enhance reaction rates by up to three orders of magnitude [31–33], we assayed the formation of **3** using the previous CH_3_CN/acetate buffer solution but containing aniline (100 mM). Under these conditions we could not detect the carbopeptide **3** even when increasing the reaction time up to 48 h. Results were improved by lyophilizing the template prior to the oxime ligation. In this case, the reaction afforded **3** with or without the presence of aniline in the solvent mixture. Best conditions involved the treatment of lyophilized Gal*p* with peptide aldehyde **4** in (1:1) CH_3_CN/acetate buffer for 4 h, followed by the addition of an extra amount of **4** dissolved in (1:1) CH_3_CN/acetate buffer containing aniline (100 mM). The reaction was completed after 2.5 h affording **3**, which was purified by RP-HPLC (90% purity, 64% yield) and characterized by mass spectrometry.

The synthesis of carbopeptide **1** was achieved by oxime ligation of cDTE, previously lyophilized, with peptide aldehyde **4** in a 1:1 solution of CH_3_CN and acetate buffer (0.1 M, pH 4.76) containing aniline (100 mM) at room temperature for 3 h. The crude mixture was purified yielding **1** in 98% purity and 42% yield. This methodology was extended to the preparation of carbopeptide **2** derived from peptide aldehyde **5** with one D-amino acid in the sequence. The reaction was followed by HPLC being completed after 3 h. After purification, carbopeptide **2** was obtained in 98% purity and 62% yield. The structure of carbopeptides **1** and **2** was confirmed by mass spectrometry.

### Biological activity

Carbopeptides **1**–**3** were tested for in vitro growth inhibition of phytopathogenic bacteria *Erwinia amylovora*, *Xanthomonas axonopodis* pv. *vesicatoria*, and *Pseudomonas syringae* pv. *syringae*, and of human pathogenic bacteria *Escherichia coli*, *Staphylococcus aureus*, *Lysteria monocytogenes*, and *Salmonella enterica* at 0.6, 1.2, 2.5, 5, 7.5, 10 and 20 μM (Table 1). The antibacterial activity of **BP100** and **BP143** was also evaluated for comparison purposes.

**Table 1:** Antibacterial activity (MIC) and cytotoxicity of parent peptides and carbopeptides.

Compound	MIC (μM)							Hemolysis^a^ (%)	

	*Ea*^b^	*Xav*^b^	*Pss*^b^	*Ec*^b^	*Sa*^b^	*Lm*^b^	*Se*^b^	50 μM	150 μM

KKLFKKILKYL-NH_2_ (**BP100**)	7.5–10	5–7.5	5–7.5	2.5–5	>20	>10	5–10	3 ± 0.1	22 ± 2.8
KKLfKKILKYL-NH_2_ (**BP143**)	2.5–5	5–7.5	2.5–5	10–20	>20	>20	5–7.5	2 ± 2.8	22 ± 2.6
(KKLFKKILKYL-C_2_H_4_N)_2_-cDTE (**1**)	2.5–5	0.6–1.2	1.2–2.5	2.5–5	10–20	5–7.5	2.5–5	100 ± 4.1	99 ± 5.2
(KKLfKKILKYL-C_2_H_4_N)_2_-cDTE (**2**)	2.5–5	2.5–5	2.5–5	1.2–2.5	10–20	7.5–10	2.5–5	96 ± 0.9	92 ± 7.2
(KKLFKKILKYL-C_2_H_4_N)_4_-Gal*p* (**3**)	1.2–2.5	0.6–1.2	0.6–1.2	1.2–2.5	>20	5–7.5	2.5–5	100 ± 18.8	100 ± 1.1

^a^Percentage hemolysis plus confidence interval. ^b^*Ea*, *Erwinia amylovora; Xav*, *Xanthomonas axonopodis* pv. *vesicatoria*; *Pss*, *Pseudomonas syringae* pv. *syringae; Ec, Escherichia coli; Sa, Staphylococcus aureus; Lm, Lysteria monocytogenes; Se, Salmonella enterica*.

Against phytopathogenic bacteria, carbopeptides **1** and **3** displayed higher activity than the parent peptide **BP100**. Carbopeptide **3**, derived from Gal*p*, was the most active with minimum inhibitory concentration (MIC) values from four- to eight-fold lower (MIC of 0.6 to 2.5 μM) than those of **BP100** (MIC of 5 to 10 μM). The cDTE-containing carbopeptide **1** was two- to eight-fold (MIC of 0.6 to 5 μM) more active than **BP100**. In contrast, carbopeptide **2** derived from cDTE and **BP143** showed similar activity to the parent peptide (MIC of 2.5 to 5 μM). Against the human pathogenic bacteria, the three carbopeptides **1**–**3** showed higher activity than the corresponding parent peptide, with *E. coli* and *S. enterica* being the most sensitive pathogens (MIC of 1.2 to 5 μM).

The toxicity of carbopeptides **1**–**3** to eukaryotic cells was also evaluated and it was determined as the ability to lyse erythrocytes in comparison to melittin. Percent hemolysis at 50 and 150 μM is included in Table 1. Results showed that the parent peptides **BP100** and **BP143** were not hemolytic at these concentrations. In contrast, carbopeptides **1**–**3** were able to lyse erythrocytes at both concentrations suggesting that peptide preassembly is critical for the hemolytic activity.

**Figure 2:**
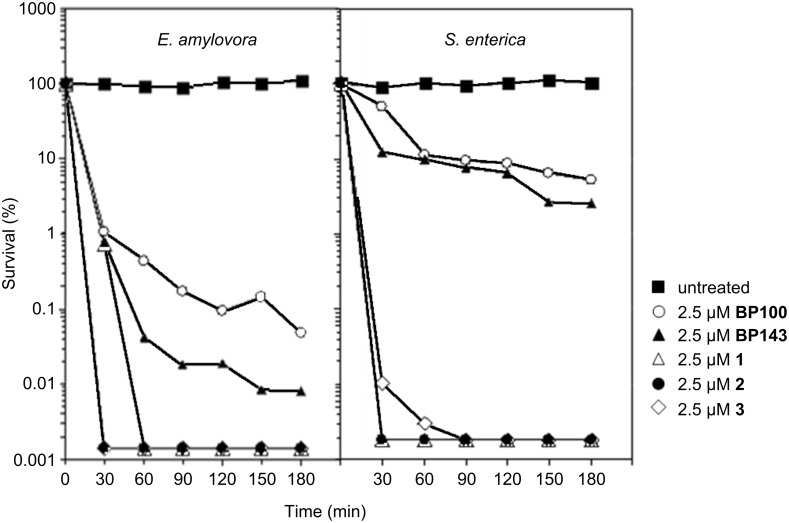
Kinetics of bactericidal activity on *E. amylovora* and *S. enterica* in the presence of peptides and carbopeptides. Viable cells were determined at different time intervals.

Moreover, we analyzed the bactericidal effect of carbopeptides **1**–**3** against *E. amylovora* and *S. enterica* by comparing the time course to kill suspensions of mid-logarithmic-phase cultures of these bacterial strains. As shown in Figure 2, the monomeric peptides **BP100** and **BP143** showed a bactericidal effect against both strains, but it was slower against *S. enterica*. Both peptides exhibited an initial fast killing stage of 30–60 min, followed by a slower rate against *E. amylovora*. Notably, carbopeptides **1**–**3** were significantly more active against both pathogens and were able to kill 99.99% of all cells within 30–90 min at 2.5 μM.

### Circular dichroism

**Figure 3:**
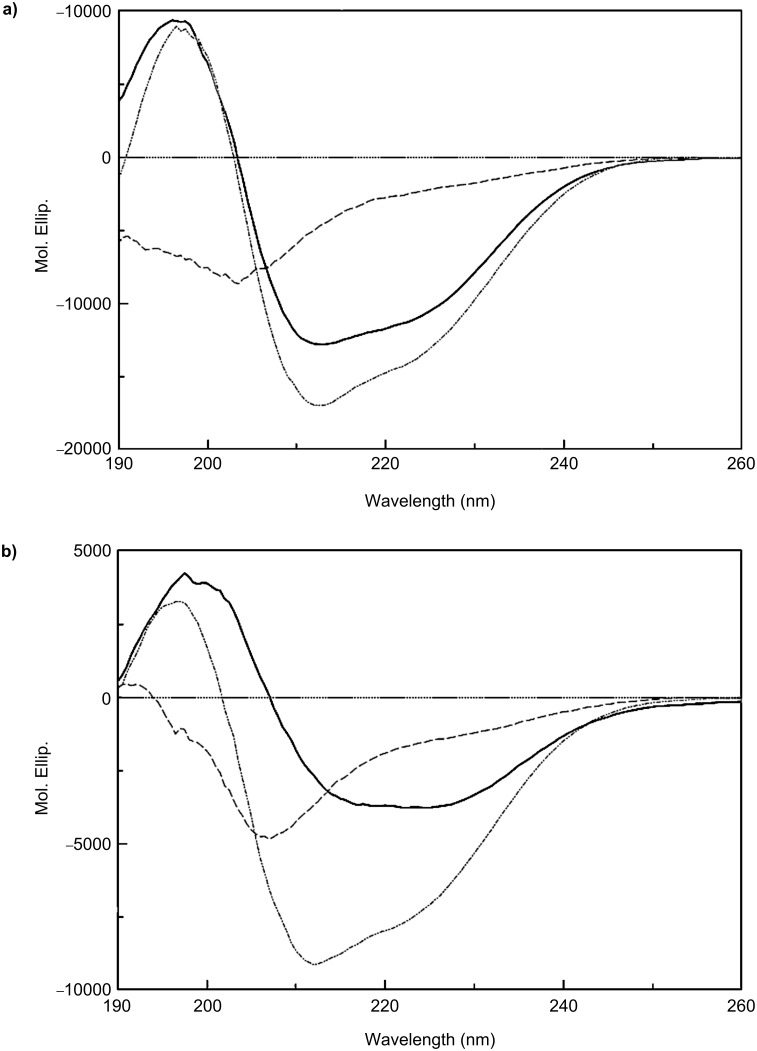
CD spectra of (a) carbopeptide **1** and of (b) carbopeptide **3** in: (i) 10 mM sodium phosphate buffer at pH 7.4 (broken line, - - -); (ii) 10 mM SDS in 10 mM sodium phosphate buffer at pH 7.4 (solid line, ^___^), and (iii) 50% (v/v) trifluoroethanol in 10 mM sodium phosphate buffer at pH 7.4 (broken dotted line, ··−··−).

We investigated the secondary structures of carbopeptides **1** and **3** by analyzing their CD spectra at 100 μM in (i) 10 mM sodium phosphate buffer at pH 7.4; (ii) 50% (v/v) trifluoroethanol (TFE) in 10 mM sodium phosphate buffer at pH 7.4; and (iii) 10 mM SDS in 10 mM sodium phosphate buffer at pH 7.4 (Figure 3). Carbopeptides **1** and **3** had similar CD spectra. In phosphate buffer they displayed a disordered structure while in SDS and in 50% TFE they became moderately structured, with the degree of secondary structure formation being higher in the latter solvent. In both cases, two local minima near 205 and 220 nm, and a positive band near 195 nm were observed. In 50% TFE, we estimated an α-helical content of 41% for carbopeptide **1** and of 19% for carbopeptide **3.** Monomeric undecapeptide **BP100** also became moderately structured in 50% TFE with an α-helical content of 21%.

## Discussion

Several studies have demonstrated that covalently tethering a number of peptides to a template can improve overall avidity for targets such as cell membranes [9–15]. In this study we used carbohydrate templates for the design of multivalent antimicrobial peptides targeted to control plant and human pathogenic bacteria and explored whether multivalency enhances the antimicrobial activity. These multivalent constructs were obtained by preassembling the antimicrobial peptides **BP100** and **BP143** to a cDTE or a Gal*p* carbohydrate template through oxime formation. They were tested against *E. amylovora*, *X. axonopodis* pv. *vesicatoria*, *P. syringae* pv. *syringae*, *E. coli*, *S. aureus*, *S. enterica*, and *L. monocytogenes*.

In agreement with previous studies, we observed that the effectiveness of the designed carbopeptides depended on the bacteria, the peptide, and the structure of the template [14–15]. For carbopeptides **1** and **2** containing two monomeric units we should expect at least a two-fold decrease in the MIC values, and for carbopeptide **3**, incorporating four peptide sequences, the activity should decrease at least four-fold. Against the phytopathogenic bacteria, carbopeptides **1** and **3**, derived from **BP100**, displayed a multimeric effect that was lower than expected. In contrast, carbopeptide **2** derived from **BP143** containing one D-amino acid did not improve the activity of the parent peptide. Against the human pathogens, we did not observe a significant decrease of the MIC values for any of the three carbopeptides. According to Kallenbach and co-workers, multivalent cationic antimicrobial peptides may display a lower activity than expected due to the potential immobilization of the cationic sequences onto the negatively charged bacterial surfaces, which may impede them to reach the bacterial membrane [15]. Similar observations were described by Shai and co-workers for covalently linked pentameric bundles of cationic antimicrobial peptides [14]. These studies demonstrated that the large volume and the charge of the bundles made their passage through the peptidoglycan or the lipopolysaccharide layers difficult, therefore preventing them from accessing the inner membrane. In contrast, despite the fact that a significant decrease of the MIC values was not observed, the preassembly of multiple copies of **BP100** and **BP143** did have an important effect on the bactericidal activity. The results showed that the carbopeptides **1**–**3** were significantly more potent than the corresponding monomers, which is in agreement with previous studies [34–35]. This ability to kill the bacteria rapidly confers them a greater capacity to control bacterial cell growth, which may be important for their future potential use as bactericidal agents.

Unlike the parent peptides **BP100** and **BP143**, carbopeptides **1**–**3** exhibited high activity towards erythrocytes. It has been shown that peptide preassembly may favor the formation of a more organized amphipathic peptide structure compared to the monomers, which has been correlated with a higher degree of hemolysis [14,36]. **BP100** was originally designed based on an ideal α-helical Edmunson wheel and showed a moderate degree of secondary-structure formation by CD. As shown by CD spectroscopy, the preassembly of **BP100** did not result in a more organized structure, especially for carbopeptide **3**, which suggests that the high hemolysis observed for carbopeptides cannot be attributed to a higher degree of secondary-structure formation. A likely explanation for the higher hemolytic activity displayed by carbopeptides could be ascribed to their different interaction with zwitterionic membranes compared to peptide monomers, as has been described in previous reports [14,36].

In summary, this study provides an example of carbopeptides with high antimicrobial activity and highlights the importance of peptide preassembly for the hemolysis and for the bactericidal activity. The structure of these carbopeptides includes two or four copies of the antimicrobial peptides **BP100** or **BP143** linked to a cDTE or a Gal*p* carbohydrate template. Even though these multivalent compounds exhibited low MIC values against plant and human pathogenic bacteria, a significant multimeric effect was not observed. Our results support the notion that preassembly of antimicrobial peptides to multimeric structures prior to contact with the microbial surface does not necessarily lead to a significant improvement of the antimicrobial activity.

## Experimental

### General methods

The synthesis of Boc_2_-Aoa-OH was described in an earlier publication from our laboratories [23]. It is now available from Polypeptide Laboratories (NeoMPS, Strassbourg, France). Automated peptide synthesis was carried out on a fully automated Syro II peptide synthesizer (MultiSynTech). Manual peptide synthesis was performed in polypropylene syringes fitted with a polyethylene porous disk. Solvents and soluble reagents were removed by suction. Chemicals were purchased from commercial suppliers Sigma–Aldrich, Fluka, NovaBiochem (Schwalbach, Germany) or Iris Biotech GmbH (Marktredwitz, Germany) and used without further purification. Microwave heating was performed in a Biotage Initiator instrument (Biotage, Uppsala, Sweden).

An acetate buffer (0.1 M, pH 4.76) was prepared by dissolving an equal amount of NaOAc·3H_2_O (10 mmol) and AcOH (10 mmol) in water (200 mL) and then adjusting to pH 4.76 with NaOH (1 M aq). An acetate buffer (0.1 M, pH 4.76) containing aniline (100 mM) was prepared by dissolving aniline (10 mmol) in the previous acetate buffer (100 mL). A UV spectrophotometer was used to quantify the amount of Fmoc cleaved.

Peptides were analyzed under standard analytical HPLC conditions with a Dionex instrument composed of an UV–vis Dionex UVD170U detector, a P680 Dionex pump, an ASI-100 Dionex automatic injector, and Chromeleon 6.60 software. Detection was performed at 220 nm. Analysis was carried out with a Kromasil 100 C_18_ (40 mm × 4.6 mm, 3.5 μm) column with a 2–100% B linear gradient over 7 min at a flow rate of 1 mL/min. Solvent A was 0.1% aq TFA, and solvent B was 0.1% TFA in CH_3_CN.

Peptide aldehydes, carbohydrate templates and carbopeptides were analyzed under standard analytical HPLC conditions with a Dionex UltiMate 3000 with Chromeleon 6.80SP3 software. Detection was performed at 215 nm. Peptide aldehydes and carbohydrate templates were analyzed with a Phenomenex Gemini 110 Å C_18_ (4.6 × 50 mm, 3 µm particle size) with a gradient of 5–80% B over 5 min and 80–100% B over 5 min (buffer A: 0.1% formic acid in H_2_O; buffer B: 0.1% formic acid in CH_3_CN) at a flow rate of 1 mL/min. Carbopeptides were analyzed on a C_4_ Phenomenex Jupiter 300 Å (4.6 × 150 mm, 5 µm particle size) with a gradient of 5–20% B over 2 min, 20–40% B over 8 min and 40–100% B over 2 min at a flow rate of 1 mL/min.

Preparative RP-HPLC was performed on a similar Dionex UltiMate 3000 system, equipped with a standard thermostatted column compartment (TCC) and with UV detection at 220 nm. Solvents used for RP-HPLC were (A) H_2_O with 0.1% TFA, (B) CH_3_CN with 0.1% TFA, (C) H_2_O and (D) CH_3_CN. A Phenomenex Gemini-NX C_18_ 110 Å column (100 × 21.20 mm, 5 µm particle size) was used, running at a flow rate of 10.0 mL/min at room temperature with gradients from 5–55% B over 24 min and 55–100% B over 11 min (method A) and at 42 °C with gradients running from 5–50% B over 24 min, 50–95% B over 11 min and 95–100% B over 2 min (method B). A FeF Chemicals C_4_ 250 Å column (20 × 250 mm, 5 μm particle size) was used running at a flow rate of 15 mL/min at 42 °C with a linear gradient of 5–95% B over 28 min (method C).

ESIMS analyses were performed on a MSQ Plus Mass Spectrometer (Thermo) or by using an Esquire 6000 ESI ion Trap LC–MS (Bruker Daltonics). High-resolution mass spectrometry (HRMS) was recorded on a Bruker MicroToF-Q MALDI instrument by using a hybrid quadrupole time-of-flight mass spectrometer (University of Zaragoza).

Circular dichroism (CD) measurements were obtained using a Jasco spectropolarimeter (J-810, Easton, MD, USA) at 25 °C. Spectra were obtained in a fused quartz cell with 1 mm path length over a wavelength range 190–260 nm at 0.1 nm intervals, 50 nm/min speed, 0.5 s response time, and 1 nm bandwidth.

### Solid-phase synthesis of peptides **BP100** and **BP143**

Peptides were synthesized manually by the solid-phase method using standard Fmoc chemistry. Fmoc-Rink-MBHA resin (0.56 mmol/g) was used as a solid support. Couplings of the corresponding amino acids Fmoc-Leu-OH, Fmoc-Phe-OH, Fmoc-D-Phe-OH, Fmoc-Lys(Boc)-OH, Fmoc-Ile-OH, and Fmoc-Tyr(*t*-Bu)-OH (4 equiv) were performed using ethyl cyanoglyoxylate-2-oxime (Oxyma) (4 equiv), *N*,*N*′-diisopropylcarbodiimide (DIPCDI) (4 equiv) in DMF at room temperature for 1 h. The completion of the reactions was monitored by the Kaiser test. Fmoc group removal was achieved with piperidine/DMF (3:7, 1 × 2 min + 1 × 10 min). After each coupling and deprotection step, the resin was washed with DMF (6 × 1 min) and CH_2_Cl_2_ (3 × 1 min) and air-dried. Once the peptide sequence had been completed, the Fmoc group was removed. Acidolytic cleavage was performed by treatment of the resin with TFA/H_2_O/TIS (95:2.5:2.5) for 2 h at room temperature. Following concentration in vacuo by evaporation of TFA and trituration with Et_2_O, the crude peptide was dissolved in H_2_O/CH_3_CN, lyophilized, analyzed by HPLC, and characterized by ESIMS.

Peptide KKLFKKILKYL-NH_2_ (**BP100**)

HPLC: *t*_R_ = 4.07 min (90% purity); ESIMS (*m*/*z*): 1420.87 [M + H]^+^.

Peptide KKLfKKILKYL-NH_2_ (**BP143**)

HPLC: *t*_R_ = 6.28 min (84% purity); ESIMS (*m*/*z*): 1420.87 [M + H]^+^.

### Solid-phase synthesis of peptide aldehydes **4** and **5**

TentaGel S NH_2_ (TG) resin (0.27 mmol/g) was placed in a syringe and swelled with CH_2_Cl_2_ (3 × 10 min) and DMF (3 × 10 min). The *ortho* backbone amide linker (*o*-BAL) was incorporated by treating the resin twice with 5-(2-formyl-3,5-dimethoxyphenoxy)pentanoic acid (4.0 equiv), *N*,*N*,*N′*,*N*′-tetramethyl-*O*-(1*H*-benzotriazol-1-yl)uronium hexafluorophosphate (HBTU) (3.8 equiv), 1-hydroxybenzotriazole (HOBt) (4.0 equiv), and *N*,*N*-diisopropylethylamine (DIPEA) (7.8 equiv) in DMF at room temperature for 12 h. Washings were performed with DMF (3 × 1 min), CH_2_Cl_2_ (3 × 1 min) and DMF (3 × 1 min). Next a reductive amination was performed by using aminoacetaldehyde dimethyl acetal (10 equiv) and NaBH_3_CN (10 equiv) suspended in AcOH/DMF (1:99). This suspension was added to the *o*-BAL-TG resin and the mixture was heated in a microwave instrument at 60 °C for 10 min. The resin was washed with DMF (3 × 1 min), CH_2_Cl_2_ (3 × 1 min) and dichloroethane (DCE) (3 × 1 min) and the reductive amination step was repeated. Then, Fmoc-Leu-OH (10 equiv) was dissolved in DCE/DMF (10:1), DIPCDI (5 equiv) was added, and after preactivation for 10 min, the slurry was transferred to the resin. The reaction mixture was heated in a microwave instrument to 60 °C for 2 × 10 min, and then the resin was washed with DMF (3 × 1 min), CH_2_Cl_2_ (3 × 1 min) and DMF (3 × 1 min). Peptide elongation was performed on the fully automatic synthesizer MultiSynTech Syro II by repeated cycles of amino acid coupling, Fmoc group removal and washings. The amino acid couplings were carried out by using the corresponding Fmoc-amino acid (6 equiv), HBTU (5.8 equiv), HOBt (6 equiv) and DIPEA (11.7 equiv) shaking the mixture for 2 h at room temperature. DMF was used as solvent for all the couplings except for Fmoc-Phe-OH and Fmoc-D-Phe-OH, which were coupled in *N*-methyl-2-pyrrolidinone (NMP). Each coupling step was repeated twice. The Fmoc group was removed by treating the resin with piperidine/DMF (2:3, 3 min) followed by two treatments of piperidine/DMF (1:4, 12 + 15 min). After coupling and deprotection steps the resin was washed with DMF (3 × 1 min), CH_2_Cl_2_ (1 × 1 min) and DMF (3 × 1 min). Once the second amino acid was coupled, a Fmoc quantification was performed to estimate the overall loss in loading.

Once the synthesis was completed, peptides were individually cleaved from the resin with TFA/H_2_O (95:5) for 2 h. Following concentration in vacuo by evaporation of TFA and trituration with Et_2_O, the crude peptides were dissolved in H_2_O, analyzed by HPLC, purified by preparative RP-HPLC using Method A and characterized by LC–MS.

Peptide aldehyde KKLFKKILKYLG-H (**4**)

HPLC: *t*_R_ = 2.08 min (99% purity); ESIMS (*m*/*z*): 366.7 [M + 4H]^4+^, 488.6 [M + 3H]^3+^.

Peptide aldehyde KKLfKKILKYLG-H (**5**)

HPLC: *t*_R_ = 2.20 min (99% purity); ESIMS (*m*/*z*): 366.6 [M + 4H]^4+^, 488.5 [M + 3H]^3+^.

### Preparation of the aminooxy-functionalized templates cDTE and Gal*p*

The synthesis of the functionalized cyclo-dithioerythritol (cDTE) and α-D-galactopyranoside (Gal*p*) templates have been described in earlier publications [23,30].

#### Synthesis of carbopeptide (KKLFKKILKYL-C_2_H_4_N)_2_-cDTE (**1**)

(4*R*,5*S*)-1,2-Dithiane-4,5-diyl bis(Boc)_2_-Aoa (cDTE) (12.7 mg, 18.2 μmol) was dissolved in TFA/CH_2_Cl_2_ (1:1, 2 mL) and stirred for 1 h. The solution was then concentrated in vacuo, redissolved in a small amount of water and lyophilized to afford (4*R*,5*S*)-1,2-dithiane-4,5-diyl bis(2-Aoa) as a white powder. This template and the peptide aldehyde **4** (65.7 mg, 45.0 μmol) were dissolved in 6.2 mL of a 1:1 solution of CH_3_CN and an acetate buffer (0.1 M, pH 4.76) containing aniline (100 mM). The reaction mixture was stirred for 3 h and purified by preparative RP-HPLC using Method B (24.3 mg, 42% yield).

HPLC: *t*_R_ = 2.50 min (98% purity); ESIMS (*m*/*z*): 1594.5 [M + 2H]^2+^, 1063.5 [M + 3H]^3+^, 797.8 [M + 4H]^4+^, 638.4 [M + 5H]^5+^, 532.2 [M + 6H]^6+^; HRMS–MALDI (*m*/*z*): [M + Na]^+^ calcd for C_156_H_264_N_36_NaO_30_S_2_, 3208.9753; found, 3208.9737.

#### Synthesis of carbopeptide (KKLfKKILKYL-C_2_H_4_N)_2_-cDTE (**2**)

(4*R*,5*S*)-1,2-Dithiane-4,5-diyl bis(2-(Boc)_2_-Aoa) (cDTE) (4.8 mg, 6.9 μmol) was dissolved in TFA/CH_2_Cl_2_ (1:1, 1 mL) and stirred for 1 h. The solution was then concentrated in vacuo, redissolved in a small amount of water and lyophilized to afford (4*R*,5*S*)-1,2-dithiane-4,5-diyl bis(2-Aoa) as a white powder. This template and the peptide aldehyde **5** (30.2 mg, 20.6 μmol) were dissolved in 1:1 solution (2 mL) of CH_3_CN and an acetate buffer (0.1 M, pH 4.76) containing aniline (100 mM). The reaction mixture was stirred for 3 h and purified by preparative RP-HPLC using Method B (13.6 mg, 62% yield).

HPLC: *t*_R_ = 2.51 min (98% purity); ESIMS (*m*/*z*): 1594.1 [M + 2H]^2+^, 1063.5 [M + 3H]^3+^, 797.9 [M + 4H]^4+^, 638.4 [M + 5H]^5+^, 532.2 [M + 6H]^6+^; HRMS–MALDI (*m*/*z*): [M + H]^+^ calcd for C_156_H_265_N_36_O_30_S_2_, 3186.9753; found, 3186.9820.

#### Synthesis of carbopeptide (KKLFKKILKYL-C_2_H_4_N)_4_-Gal*p* (**3**)

Methyl 2,3,4,6-tetra-*O*-((Boc)_2_-Aoa)-α-D-Gal*p* (3.4 mg, 2.6 μmol) was dissolved in TFA/CH_2_Cl_2_ (1:1, 0.5 mL) and stirred for 30 min at room temperature. The solution was then concentrated to dryness, redissolved in a small amount of water and lyophilized to afford methyl 2,3,4,6-tetra-*O*-Aoa-α-D-Gal*p* as a white powder. This lyophilized template was dissolved in 1.2 mL of a 1:1 solution of CH_3_CN and an acetate buffer (0.1 M, pH 4.76). Peptide aldehyde **4** (19.3 mg, 13.2 μmol) was added, and the mixture was stirred at room temperature. The reaction was followed by HPLC. After 4 h, an additional amount of peptide aldehyde **4** (20 mg, 13.7 μmol) dissolved in 1.1 mL of a 1:1 solution of CH_3_CN and an acetate buffer (0.1 M, pH 4.76) containing aniline (100 mM) was added. After further 2.5 h, the reaction was completed. The reaction mixture was purified by RP-HPLC using Method C (10.7 mg, 64% yield).

HPLC: *t*_R_ = 6.23 min (90% purity); ESIMS *m*/*z*: 1566.3 [M + 4H]^4+^, 1253.8 [M + 5H]^5+^, 1045.1 [M + 6H]^6+^, 895.7 [M + 7H]^7+^, 784.2 [M + 8H]^8+^, 697.2 [M + 9H]^9+^, 627.5 [M + 10H]^10+^ ([M]^+^ calcd for C_311_H_526_N_72_O_62_, 6262.022 Da).

### Bacterial strains and growth conditions

As described in [28], the following plant pathogenic and foodborne bacterial strains were used: *Erwinia amylovora* PMV6076 (Institut National de la Recherche Agronomique, Angers, France), *Pseudomonas syringae* pv. *syringae* EPS94 (Institut de Tecnologia Agroalimentària, Universitat de Girona, Spain), and *Xanthomonas axonopodis* pv. *vesicatoria* 2133-2 (Instituto Valenciano de Investigaciones Agrarias, Valencia, Spain). Moreover, the following bacterial strains were also used: *Escherichia coli* ATCC NCTC 5934, *Listeria monocytogenes* ATCC 15313, *Staphylococcus aureus* subsp. *aureus* ATCC 9144 and *Salmonella enterica* subsp. *enterica* LT2 ATCC 15277.

All bacteria were stored in Luria Bertani (LB) broth except for *L. monocytogenes*, which was stored in Brain Heart Infusion (BHI) broth (Oxoid, Hampshire, United Kingdom) supplemented with glycerol (20%) and maintained at −80 °C. *E. amylovora* and *P. syringae* pv*. syringae* were scrapped from LB agar after growing for 24 h at 25 °C; *X. axonopodis* pv. *vesicatoria* was scrapped from LB agar after growing for 48 h at 25 °C; *S. aureus, E. coli* and *S. enterica* were scrapped from LB agar after growing for 24 h at 37 °C; *L. monocytogenes* was scrapped from BHI agar after growing for 24 h at 37 °C. The cell material was suspended in sterile water to obtain a suspension of 10^8^ CFU mL^−1^.

### Antibacterial activity

As described in [28], lyophilized compounds were solubilized in sterile Milli-Q water to a final concentration of 1000 μM and filter sterilized through a 0.22 μm pore filter. For minimum inhibitory concentration (MIC) assessment, dilutions of the compounds were made to obtain a final concentration of 200, 100, 75, 50, 25, 12.5 and 6 μM. Twenty microliters of each dilution were mixed in a microtiter plate well with 20 μL of the corresponding suspension of the bacterial indicator, 160 μL of Trypticase Soy Broth (TSB) (BioMèrieux, France) for *E. amylovora, P. syringae* pv*. syringae, X. axonopodis* pv. *vesicatoria, S. aureus, E. coli* and *S. enterica* or BHI for *L. monocytogenes* to a total volume of 200 μL. Three replicates for each strain, compound and concentration were used. Positive controls contained water instead of compound, and negative controls contained compounds without bacterial suspension. Microbial growth was automatically determined by optical density measurement at 600 nm (Bioscreen C, Labsystem, Helsinki, Finland). Microplates were incubated at 25 °C for plant pathogenic bacteria and at 37 °C for foodborne bacterial strains, with 20 s shaking before hourly absorbance measurement, for 48 h. The experiment was repeated twice. The MIC was taken as the lowest compound concentration with no growth at the end of the experiment.

### Bactericidal activity

The bactericidal activity was determined for peptides **BP100** and **BP143** and for carbopeptides **1**–**3** by using *E. amylovora* and *S. enterica* as indicators of plant pathogenic and foodborne bacteria. Ringer bacterial suspensions at 1–1.5 × 10^6^ CFU mL^−1^ were incubated in a 2.5 µM concentration of the corresponding compound, including also a negative control containing only Ringer solution. A total volume of 13 mL suspension was incubated for 3 h at room temperature. Aliquots of 1.5 mL were removed at 30 min intervals, tenfold-diluted if necessary, and spread in triplicate onto LB agar plates by using a spiral plater (Eddy Jet; IUL Instruments, Spain). After 24 h of incubation at 25 °C for *E. amylovora*, and 37 °C for *S. enterica*, the concentrations of the suspensions were obtained with an automatic counter (Flash and Go; IUL Instruments, Spain) by incorporating the software Counter Mat version 5.0. Values are expressed as percentages of survival from the start of the experiment.

### Hemolytic activity

As described in [28], the hemolytic activity of the compounds was evaluated by determining hemoglobin release from erythrocyte suspensions of fresh human blood (5% vol/vol). Blood was aseptically collected using a BD vacutainer K2E System with EDTA (Belliver Industrial State, Plymouth, U.K.) and stored for less than 2 h at 4 °C. Blood was centrifuged at 6000*g* for 5 min, washed three times with tris(hydroxymethyl)aminomethane (TRIS) buffer (10 mM TRIS, 150 mM NaCl, pH 7.2) and diluted. Compounds were solubilized in TRIS buffer to a final concentration of 500, 300 and 100 μM. Fifty microliters of human red blood cells were mixed with 50 μL of the compound solution and incubated under continuous shaking for 1 h at 37 °C. Then, the tubes were centrifuged at 3500*g* for 10 min. Eighty microliter aliquots of the supernatant were transferred to 100-well microplates (Bioscreen) and diluted with 80 μL of Milli-Q water. Hemolysis was measured as the absorbance at 540 nm with a Bioscreen plate reader. Complete hemolysis was determined in TRIS buffer plus melittin at 100 µM (Sigma–Aldrich, Madrid, Spain) as a positive control. The percentage of hemolysis (*H*) was calculated by using the following equation: *H* = 100 × [(*O*_p_−*O*_b_)/(*O*_m_−*O*_b_)], where *O*_p_ was the density for a given compound concentration, *O*_b_ for the buffer, and *O*_m_ for the melittin positive control.

### Circular dichroism spectroscopy

Carbopeptides were dissolved to 100 μM concentration in 10 mM sodium phosphate buffer at pH 7.4, in 50% (v/v) TFE in 10 mM sodium phosphate buffer at pH 7.4, and in 10 mM SDS in 10 mM sodium phosphate buffer at pH 7.4. A baseline correction was made with only solvent in the cell. Data were expressed in terms of mean residue ellipticity [θ] (deg cm^2^ dmol^−1^), calculated per mol of total amide groups present in the different peptides. The percentage helicity of the peptide was calculated as follows: α-helix (%) = 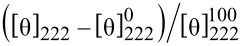, where [θ]_222_ is the experimentally observed absolute mean residue ellipticity at 222 nm. Values for 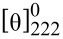 and 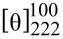, corresponding to 0 and 100% helix content at 222 nm, were estimated to be −2000 and −30000 deg cm^2^ dmol^−1^, respectively [37].

## Supporting Information

File 1 HPLC, ESIMS of peptide aldehydes **4** and **5**. HPLC, ESIMS, and HRMS of carbopeptides **1**–**3**.
