# Supplementary material for: Multivalent display of the antimicrobial peptides BP100 and BP143
Source: Beilstein J Org Chem. 2012 Dec 3;8:2106–17. doi: 10.3762/bjoc.8.237 (PMC3520567; doi:10.3762/bjoc.8.237)

Supporting Information

for

Multivalent display of the antimicrobial peptides BP100 and BP143

Imma Güell[1,‡] , Rafael Ferre[1,‡], Kasper K. Sørensen[2,‡], Esther Badosa[3], Iteng Ng-Choi[1], Emilio Montesinos[3], Eduard Bardají[1], Lidia Feliu[1], Knud J. Jensen[2,]*, Marta Planas[1,]*

Address: [1]LIPPSO, Department of Chemistry, University of Girona, Campus Montilivi, 17071 Girona, Spain, [2]IGM, Faculty of Life Sciences University of Copenhagen, DK-1871 Frederiksberg, Denmark and [3]Laboratory of Plant Pathology, Institute of Food and Agricultural Technology-CIDSAV-CeRTA, University of Girona, Campus Montilivi, 17071 Girona, Spain

Email: Knud J. Jensen - kjj@life.ku.dk; Marta Planas - marta.planas@udg.edu
*Corresponding author
‡Equally contributing authors

HPLC, ESIMS of peptide aldehydes **4** and **5**.

HPLC, ESIMS, and HRMS of carbopeptides **1**–**3**

Table of contents

Peptide aldehyde KKLFKKILKYLG-H (**4**) S2

Peptide aldehyde KKLfKKILKYLG-H (**5**) S3

(KKLFKKILKYL-$C_2H_4N)_2$-cDTE (**1**) S4

(KKLfKKILKYL-$C_2H_4N)_2$-cDTE (**2**) S6

(KKLFKKILKYL-$C_2H_4N)_4$-Gal*p* (**3**) S8

Peptide aldehyde KKLFKKILKYLG-H (4)

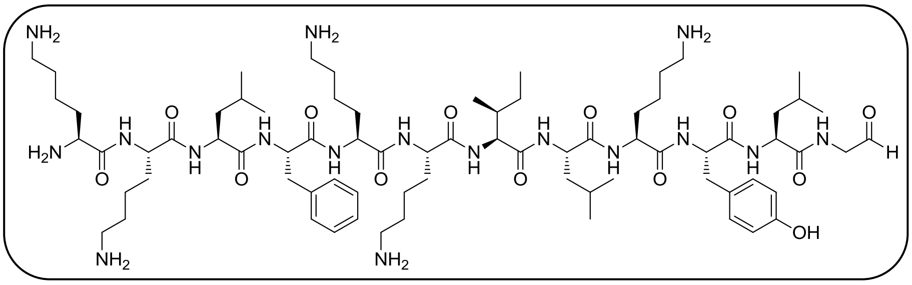

HPLC (λ = 220 nm)

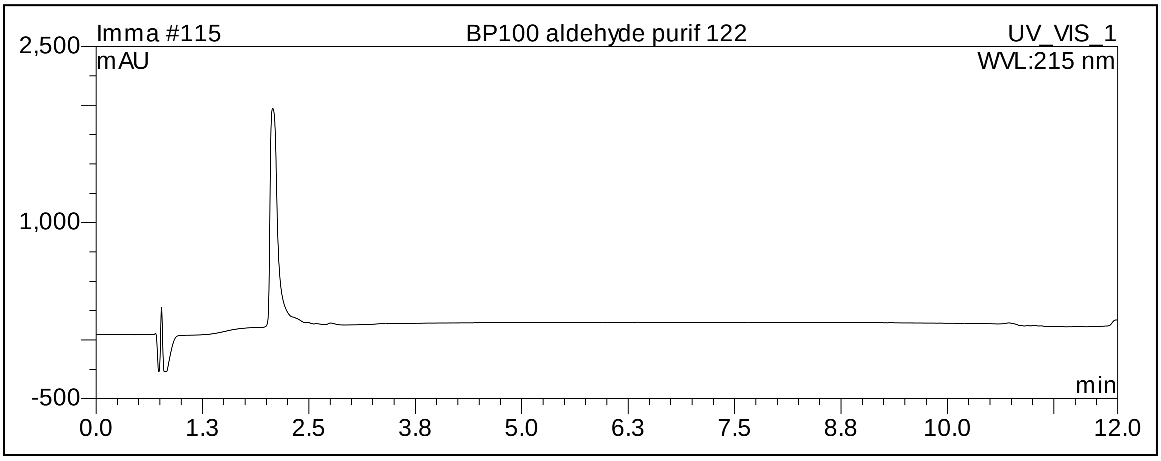

ESIMS (*m/z*)

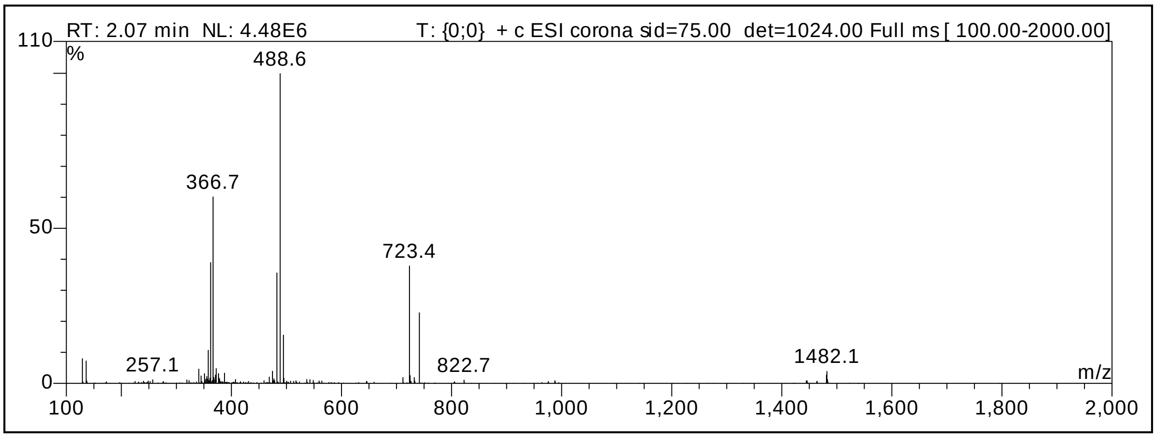

Peptide aldehyde KKLfKKILKYLG-H (5)

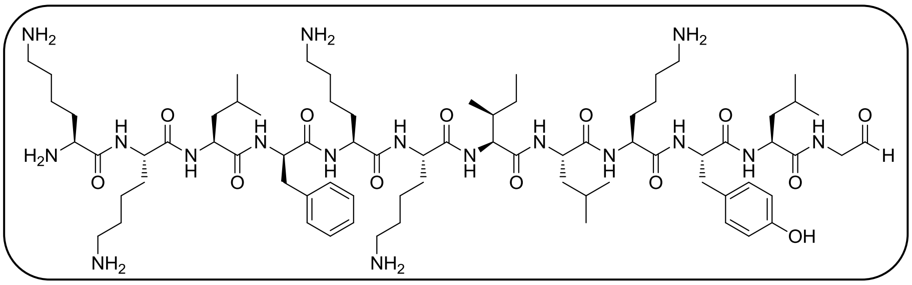

HPLC (λ = 220 nm)

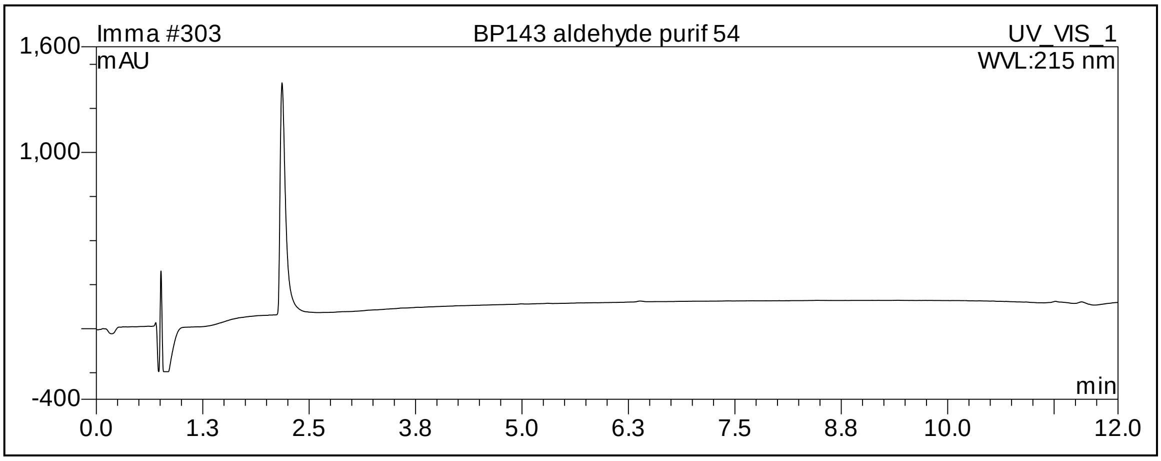

ESIMS (*m/z*)

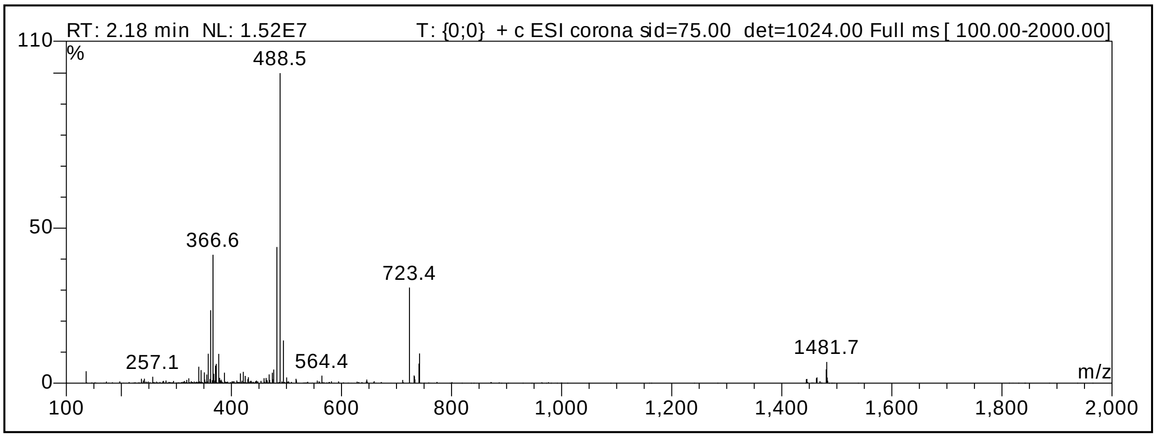

(KKLFKKILKYL-C_2H_4N)$_2$-cDTE (1)

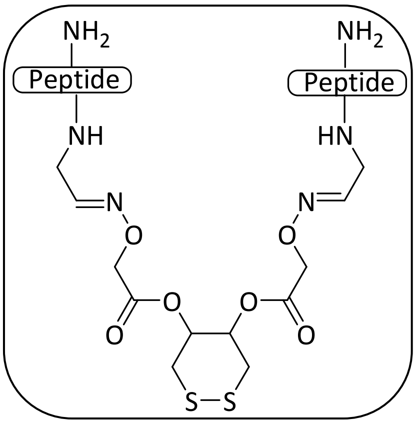

Peptide = KKLFKKILKYL

HPLC (λ = 220 nm)

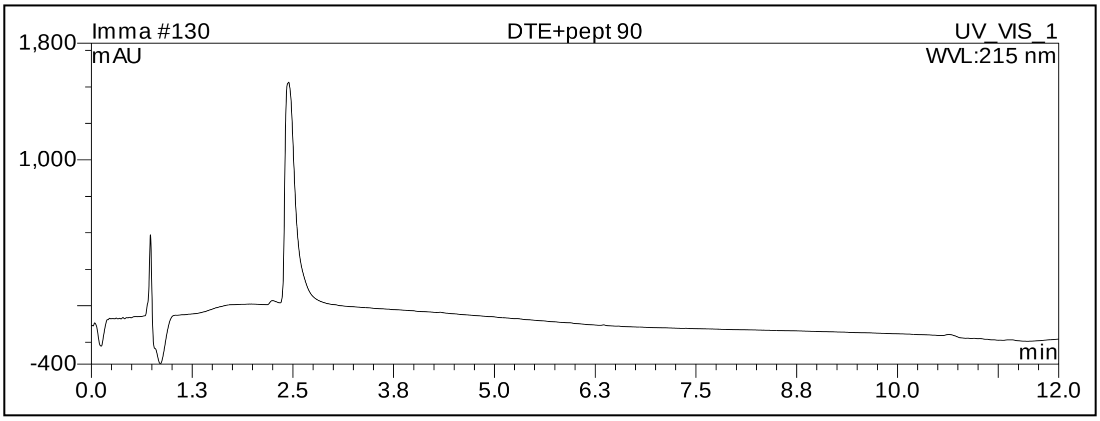

ESIMS (*m/z*)

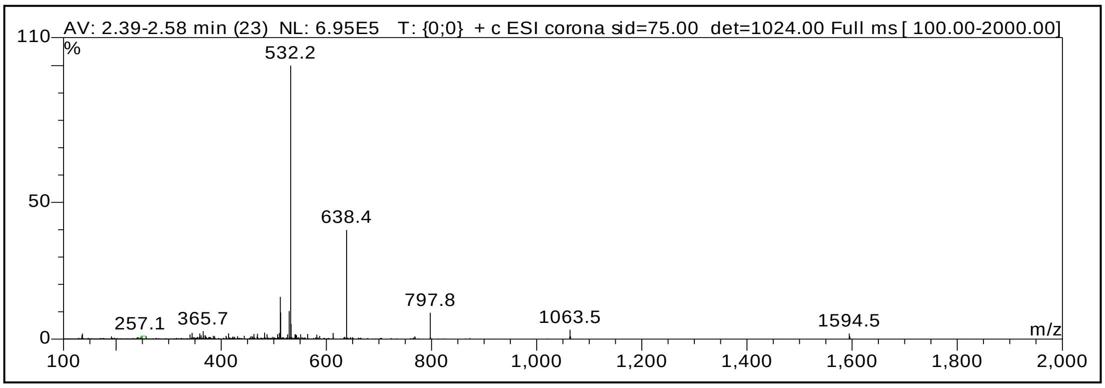

HRMS–MALDI (*m*/*z*)

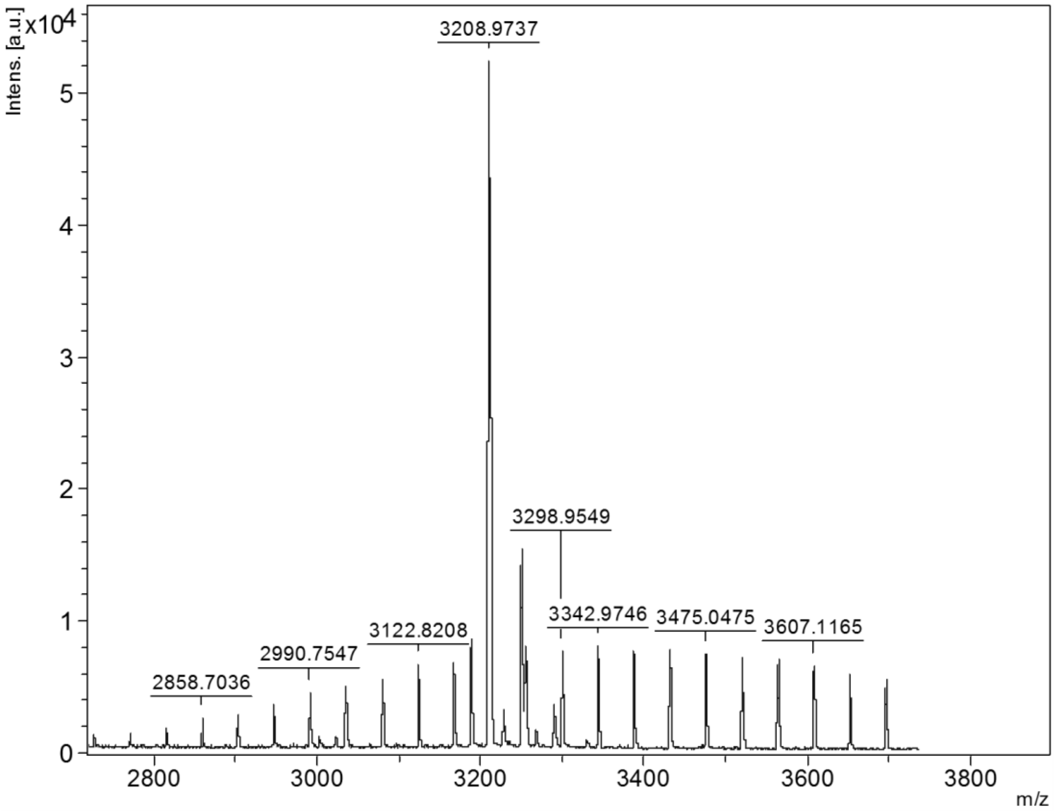

(KKLfKKILKYL-$C_2H_4N)_2$-cDTE (2)

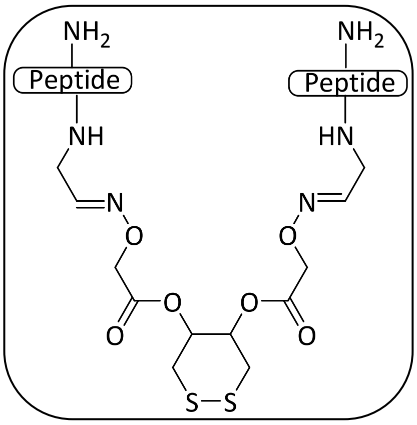

Peptide = KKLfKKILKYL

HPLC (λ = 220 nm)

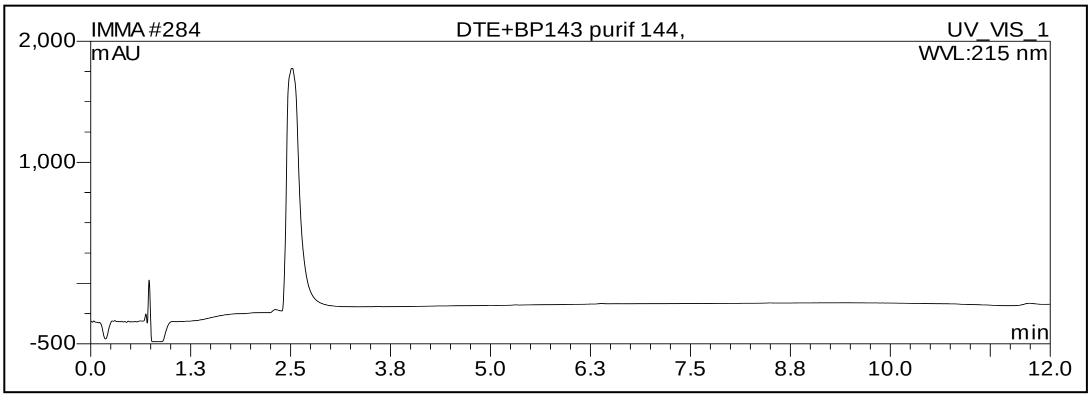

ESIMS (*m*/*z*)

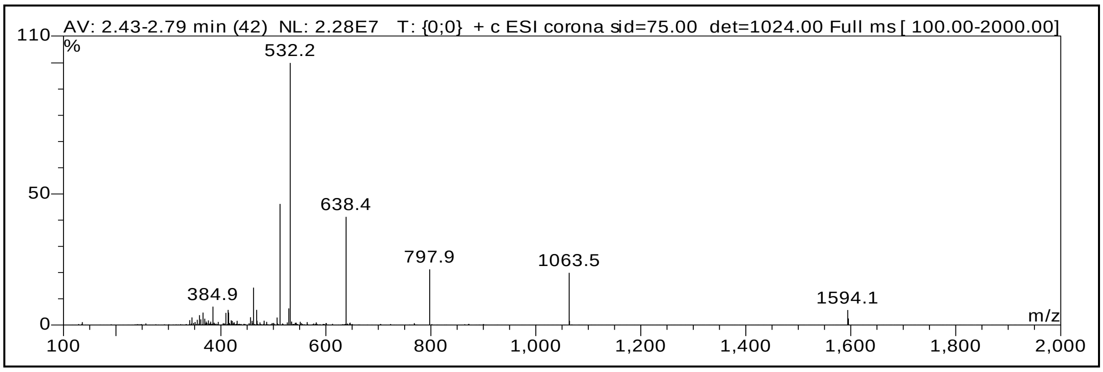

HRMS–MALDI (*m/z*)

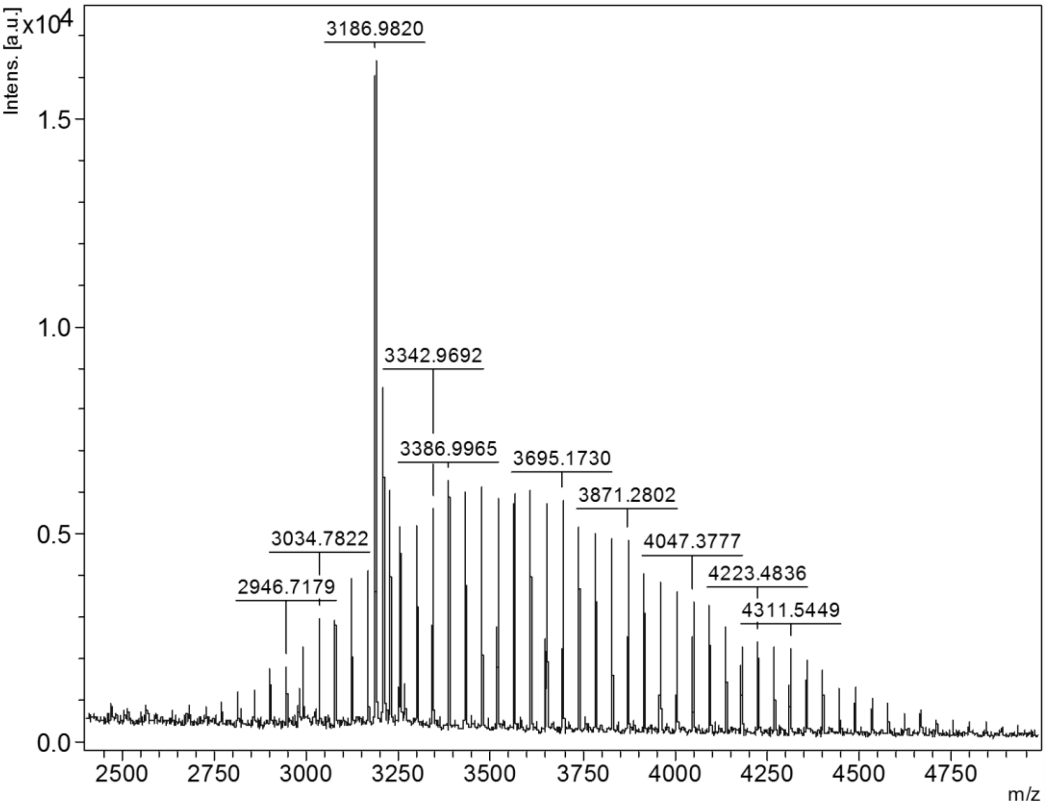

(KKLFKKILKYL-C_2H_4N)$_4$-Gal*p* (3)

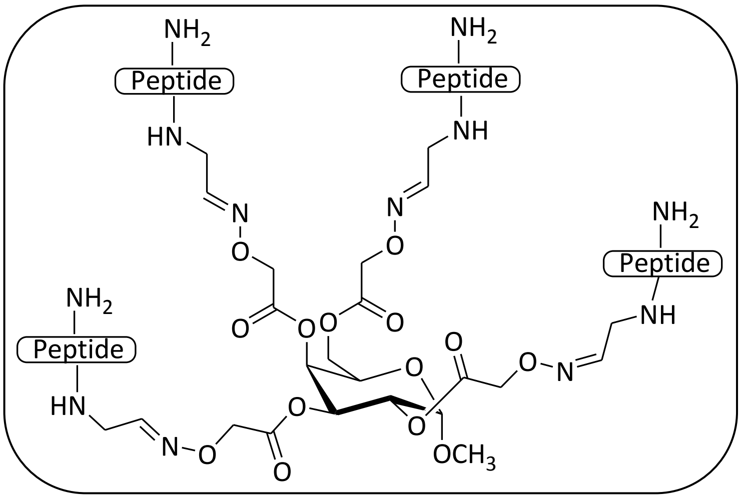

Peptide = KKLFKKILKYL

HPLC (λ = 220 nm)

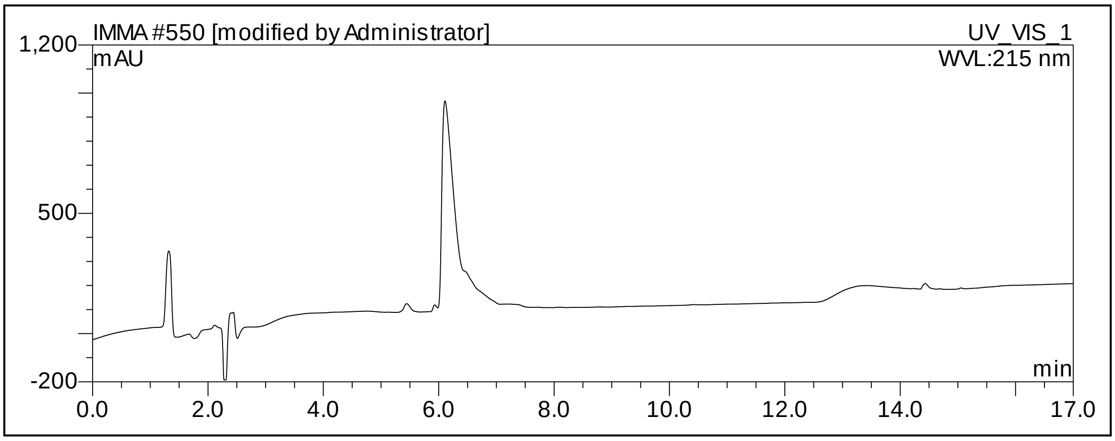

ESIMS (*m/z*)

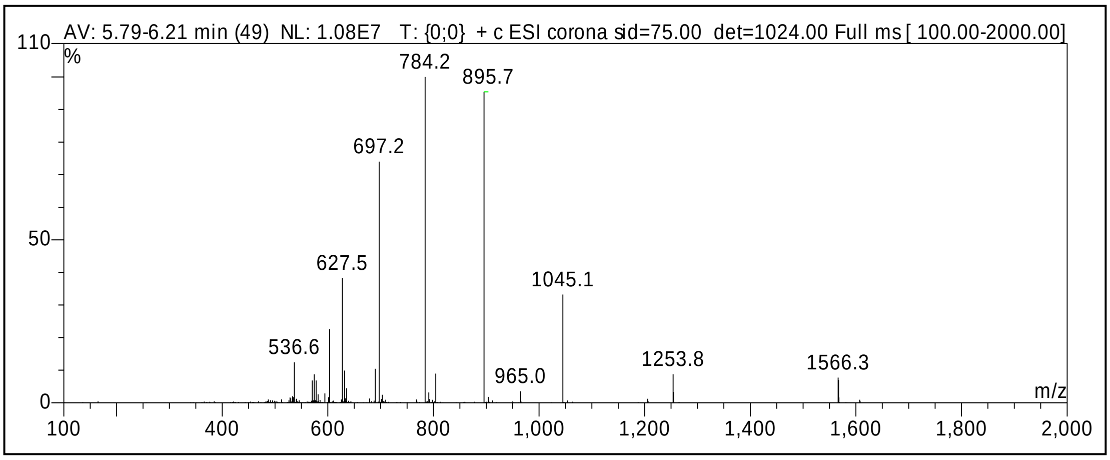

Supplement: File 1 — HPLC, ESIMS of peptide aldehydes 4 and 5. HPLC, ESIMS, and HRMS of carbopeptides 1–3. [file Beilstein_J_Org_Chem-08-2106-s001.pdf]
